# Historic and contemporary biogeographic perspectives on range‐wide spatial genetic structure in a widespread seagrass

**DOI:** 10.1002/ece3.9900

**Published:** 2023-03-19

**Authors:** Elizabeth A. Sinclair, Renae K. Hovey, Siegfried L. Krauss, Janet M. Anthony, Michelle Waycott, Gary A. Kendrick

**Affiliations:** ^1^ School of Biological Sciences University of Western Australia Crawley Western Australia Australia; ^2^ Oceans Institute, University of Western Australia Crawley Western Australia Australia; ^3^ Kings Park Science, Department of Biodiversity Conservation and Attractions Kings Park Western Australia Australia; ^4^ School of Biological Sciences University of Adelaide and State Herbarium of South Australia Adelaide South Australia Australia

**Keywords:** IMCRA bioregions, marine biogeography, microsatellite DNA, population genetics, *Posidonia australis*, predictive distribution modeling

## Abstract

Historical and contemporary processes drive spatial patterns of genetic diversity. These include climate‐driven range shifts and gene flow mediated by biogeographical influences on dispersal. Assessments that integrate these drivers are uncommon, but critical for testing biogeographic hypotheses. Here, we characterize intraspecific genetic diversity and spatial structure across the entire distribution of a temperate seagrass to test marine biogeographic concepts for southern Australia. Predictive modeling was used to contrast the current *Posidonia australis* distribution to its historical distribution during the Last Glacial Maximum (LGM). Spatial genetic structure was estimated for 44 sampled meadows from across the geographical range of the species using nine microsatellite loci. Historical and contemporary distributions were similar, with the exception of the Bass Strait. Genetic clustering was consistent with the three currently recognized biogeographic provinces and largely consistent with the finer‐scale IMCRA bioregions. Discrepancies were found within the Flindersian province and southwest IMCRA bioregion, while two regions of admixture coincided with transitional IMCRA bioregions. Clonal diversity was highly variable but positively associated with latitude. Genetic differentiation among meadows was significantly associated with oceanographic distance. Our approach suggests how shared seascape drivers have influenced the capacity of *P. australis* to effectively track sea level changes associated with natural climate cycles over millennia, and in particular, the recolonization of meadows across the Continental Shelf following the LGM. Genetic structure associated with IMCRA bioregions reflects the presence of stable biogeographic barriers, such as oceanic upwellings. This study highlights the importance of biogeography to infer the role of historical drivers in shaping extant diversity and structure.

## Graphical abstract

We combined predictive mapping with population genetic data to test two biogeographic hypotheses in a widespread temperate seagrass, *Posidonia australis*. The combined approach shows how historical and contemporary seascape drivers have influenced spatial genetic structure in *P. australis* which can effectively track sea level changes associated with natural climate cycles.

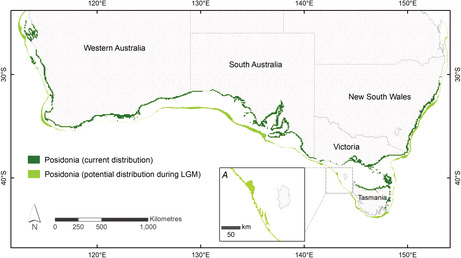

## 1 INTRODUCTION

A fundamental objective in molecular ecology is to understand how historical and contemporary processes affect patterns of diversity and contemporary distributions within and among species (Chase, 2012; Stevens, 2006; Vellend et al., 2014). Teasing apart the contribution of the biotic and abiotic drivers of genetic diversity and its spatial structuring is a significant challenge, as they interact over multiple spatiotemporal scales (Benton, 2009; Riginos et al., 2019). Insight requires an approach that combines knowledge of historical and contemporary landscape features (Galindo et al., 2006) and climate change impacts (Doney et al., 2012; Munday et al., 2013; Poloczanska et al., 2013), with population genetic tools and analyses (e.g., Assis et al., 2022; Miller et al., 2013; Selkoe et al., 2010).

**FIGURE 1:**
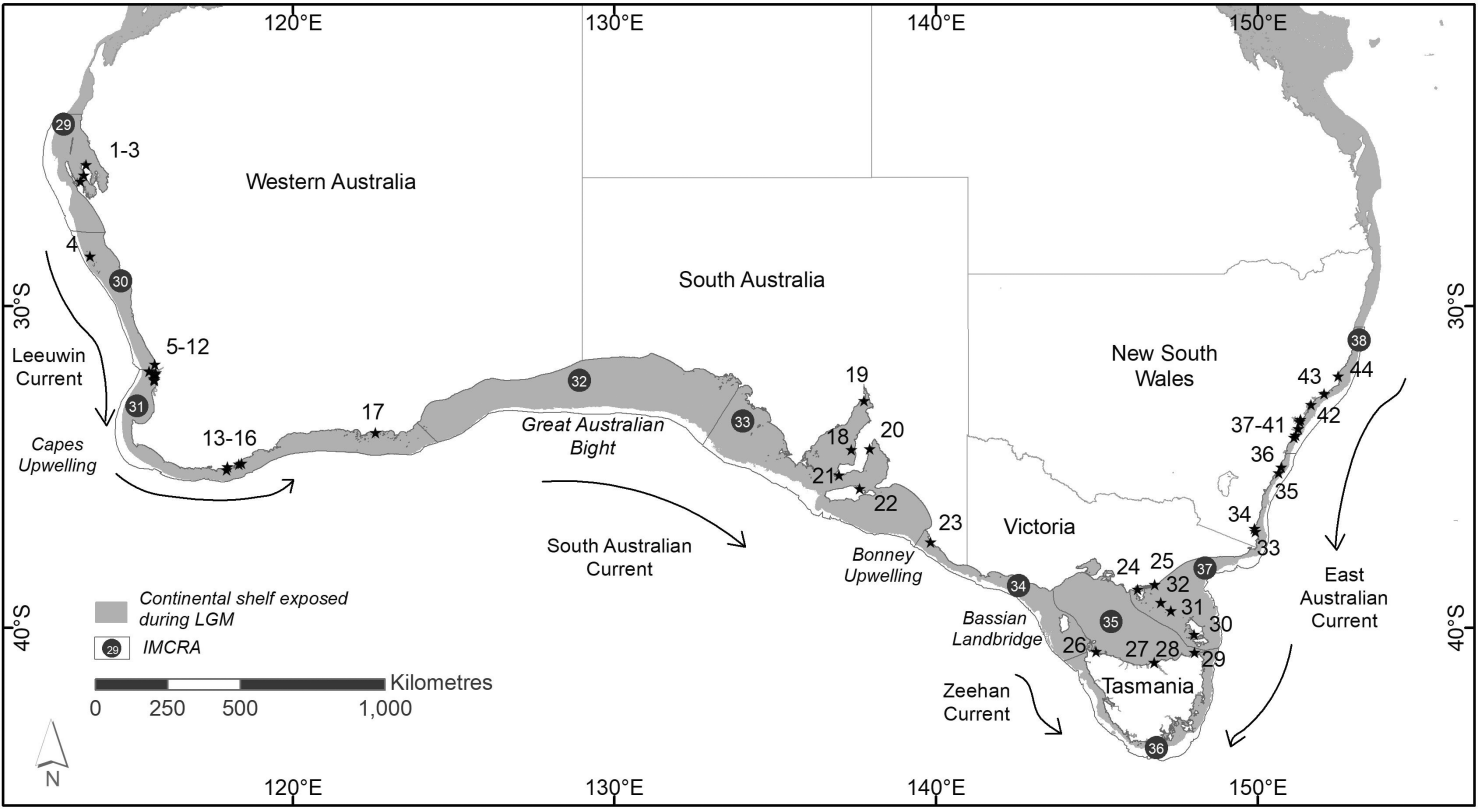
Map of Australia showing the location of sampled *Posidonia australis* meadows from across the species range around Australia. Sampled meadows are numbered (1–44): Western Australia: Guichenault (1), Useless Loop (2), Denham (3), Wallabi Island, Houtman‐Abrolhos (4), Lal Bank, Marmion Marine Park (5), Fremantle fishing boat harbor (6), Parker Point, Rottnest Island (7), Parmelia Bank (8), Carnac Island (9), Southern Flats, Cockburn Sound (10), Point Peron (11), Safety Bay (12), Whalers Cove, Frenchman's Bay (13), Oyster Harbour (14), Waychinicup Inlet (15), Cheynes Beach (16), Duke of Orleans Bay, Esperance (17); South Australia: Goose Island, Spencer Gulf (18), Fitzgerald Bay, Spencer Gulf (19), Ardrossan (20), Marion Bay (21), American River, Kangaroo Island (22), Nora Creina (23); Victoria: Saint Margaret Island (24) and Duck Point (25), Corner Inlet; Tasmania: Stony Point, Robbins Passage (26), Sea Oak Point, Tamar River (27), Low Head, Tamar River (28), Little Musselroe Bay (29), Fotheringate Beach, Flinders Island (30), Deal Island, Kent Group (31), Hogan Island (32); New South Wales: Pambula Lake (33), Merimbula Lake (34), St Georges Basin (35), Jervis Bay (36), Port Hacking (37), Botany Bay (38), Port Jackson (Sydney Harbour, 39), Pittwater (40), Brisbane Water (41), Lake Macquarie (42), Port Stephens (43), and Wallis Lake (44). The boundary currents are indicated, along with potential barriers to gene flow: Capes Upwelling, Great Australian Bight, Bonney Upwelling, and Bassian Landbridge. Continental shelf is shown in dark gray. Numbers in black circles (29–38) are inshore coastal provincial bioregions (based on Integrated Marine and Coastal Regionalisation of Australia; IMCRA): (29) central western, (30) southwest transition, (31) southwest, (32) Great Australian Bight transition, (33) Spencer Gulf, (34) western Bass Strait transition, (35) Bass Strait, (36) Tasmanian, (37) southeast transition and (38) central eastern.

Key biogeographic features have impacted Australian coastal shelf waters since the emergence of marine flowering plants (100–70 mya). The passive continental shelf margins surrounding the Australian continent evolved through a series of seafloor‐spreading episodes 120–55 mya that created habitat for coastal marine plants (Falvey & Mutter, 1981). Ocean current dynamics around southern Australia were established during the Miocene (Gallagher et al., 2001), with the formation of major boundary currents that include the Leeuwin Current, South Australian Current/Zeehan Current, and the East Australian Current (Figure 1; James & Bone, 2011). Upwelling zones include the Capes Current in Western Australia (Gersbach et al., 1999; Pearce & Pattiaratchi, 1999) and the Bonney Upwelling in southeastern Australia (Kämpf et al., 2004). Glacially mediated sea level changes affected the extent of continental shelf habitat available, where habitats were eliminated or significantly reduced, and reformed and expanded over time (Dolby et al., 2016). The lowest recorded sea level at ~120 m below present occurred during the Last Glacial Maximum (LGM) between 21,000 and 19,000 years BP (Lewis et al., 2013), leaving most of Australia's current continental shelf exposed, and a land bridge connecting mainland Australia to Tasmania. Shoreline reconstructions show marine inundation of the Bassian Landbridge began ~17,500 years ago from the west (figure 4 in Lambeck & Chappell, 2001). Drowned (submerged) shorelines persist (Brooke et al., 2017), leaving a paleorecord as rising sea levels inundated the shelf. Independent verification using carbon dating of *P. australis* sheath remains from seafloor sedimentary cores in western and central‐southern Australia, estimated at up to 3500 years old (Serrano et al., 2016), is consistent with recolonization of inshore coastal environments post‐LGM. However, these clues provide no insight as to whether recolonization occurred from discrete refugia or more widespread edge‐of‐shelf meadows.

Three broad biogeographic provinces are currently recognized for marine biota across the temperate Australian coastline: Flindersian (west and south coasts), Maugean (Bass Strait), and Peronian (east coast) (Bennett & Pope, 1953; Waters et al., 2010). These are based on species turnover, and a combination of extrinsic (water movement and historical barriers) and intrinsic (life history and habitat type) factors that explain why these relationships largely hold across a range of taxa (e.g., Ayre et al., 2009; Li et al., 2013; York et al., 2008), despite the absence of barriers, such as the Bassian Landbridge (figure 2 in Williams et al., 2018). These broad‐scale provinces are, however, likely to miss biologically meaningful barriers that have caused significant genetic structure. In response, Waters et al. (2010) recommended a quantitative approach to testing finer‐scale, regional marine biogeographical frameworks. An Integrated Marine and Coastal Regionalisation Framework for Australian waters (IMCRA v4.0; Commonwealth of Australia, 2006; Last et al., 2010) was developed to capture spatial patterns in species distributions. The IMCRA framework defined inshore coastal provincial bioregions based on demersal fish biogeography, of which 10 regions span southern Australia (Figure 1). Six are classified as regions of “biotic endemism” and include subtropical, warm temperate, or cool temperate waters, and four are identified as “transitions” which are less well‐defined mixing areas. This regional framework can be used to classify populations and communities into bioregions that make ecological sense (e.g., reflect connectivity) and are at a scale useful for regional planning. A multispecies approach by Pope et al. (2015) determined that when latitudinal information was combined with the IMCRA model for 101 species, genetic diversity in populations increased toward the equator, although a notable “hump” with high genetic diversity was observed at the temperate–tropical zone along the Western Australian coastline (IMCRA 29; Pope et al., 2015). Here, we test whether these bioregions are biologically meaningful for a widespread temperate benthic habitat‐forming seagrass species, *Posidonia australis*.

The genus *Posidonia* belongs to an ancient group of marine angiosperms, estimated to have diverged more than 50 mya, based on molecular fossil‐dated phylogenies (Waycott et al., 2018). The extant Australian members of the genus began diverging approximately 12.8 mya (Aires et al., 2011) and possess similar biological attributes including a perennial habit capable of forming clonal plants with potential for extreme longevity and size (e.g., Arnaud‐Haond et al., 2012; Edgeloe et al., 2022). Ribbon weed, *Posidonia australis* Hook.f., is endemic to the temperate waters of southern Australia (Edgar, 2000). Its distribution spans approximately 5300 km of coastline south from Shark Bay in the temperate–tropical zone in Western Australia to Wallis Lake in central New South Wales. Distribution records show the species is naturally disjunct across its range, and increasing anthropogenic impacts have caused significant declines (Evans et al., 2018; Short et al., 2011). This species typically grows in large continuous meadows from the low tide mark to 15 m depth, favoring more sheltered bays and estuaries (Cambridge & Kuo, 1979; Carruthers et al., 2007). Fruit production is highly variable across the species' range, but often prolific in the southwest of Australia. The fruit, containing a single direct‐developing seed with no dormancy, begins releasing from lower latitudes in the austral spring (November–January; Kuo & McComb, 1989). Positively buoyant fruit are dispersed on the sea surface via currents and windage (Ruiz‐Montoya et al., 2012), frequently covering distances of 10 s of km over a few days (Ruiz‐Montoya et al., 2015; Sinclair et al., 2018).

We modeled the historical and contemporary distributions of *P. australis* and assessed range‐wide genetic diversity using microsatellite DNA data to address the following questions: (1) How is genetic diversity spatially structured among meadows sampled from across the 5300 km range of this species; (2) Does this spatial genetic structure correspond to the broad‐ and fine‐scale biogeographical models for the temperate Australian continental shelf; and (3) Were historical meadows widespread or restricted in isolated refugia during the LGM?

## 2 MATERIALS AND METHODS

### 2.1 Field sampling and genotyping

This study includes collections made from across the species’ entire geographic range between 2004 and 2016. These meadows covered the three biogeographic provinces and 8 of 10 IMCRA bioregions (Table 1; Figure 1). Thirty individual shoots from each of the 44 *P. australis* meadows were sampled following methods described by Sinclair et al. (2014). DNA was extracted from meristem tissue and genotyped for nine polymorphic microsatellite loci (*Pa*A1, *PaA*105, *Pa*A120, *Pa*B6, *Pa*B8, *Pa*B112, PaD12, *Pa*D113, and *Pa*118/9), using methods previously described (Sinclair et al., 2009, 2014). We combined data from published regional studies (Evans et al., 2014; Sinclair et al., 2014, 2016) with sampling from an additional 14 meadows for a range‐wide assessment. A subset of samples were used as positive controls in each run to ensure consistent scoring of alleles across locations. There was no widespread evidence for linkage disequilibrium, presence of null alleles, stuttering, or large allele dropout from our previous studies, as assessed using MICRO‐CHECKER (van Oosterhout et al., 2004).

**TABLE 1:** *Posidonia australis* meadows sampled throughout southern Australia, by state, biogeographic province, and IMCRA bioregion and genetic diversity indices.

No.	Sample site (state/province)	Abbrev.	IMCRA bioregion	Depth (m)	Latitude (S)	Longitude (E)	*N*	MLG	*R*	Na	*p*[*i*]	*H*o (%)	*H*e (%)	*F*	3×
Western Australia (Flindersian)															
1	Guichenault Point, Shark Bay	GU	29	0.5	25° 35′ 58.3″	113° 34′ 53.2″	30	15	0.48	24	–	25.9	25.5	0.035	1
2	Useless Loop, Shark Bay	UL	29	3.0	26° 06′ 37.8″	113° 24′ 19.3″	30	7	0.21	19	–	61.9	35.8	−0.666**	23
3	Denham, Shark Bay	DE	29	1.0	25° 55′ 12.2″	113° 30′ 52.4″	30	18	0.59	27	1	56.2	38.3	−0.373**	25
4	Wallabi Island, Abroholis Islands	AB	30	1.0	28° 26′ 27.2″	113° 42′ 11.8″	30	1	0.00	16	–	66.7	33.3	−1.000**	30
5	Lal Bank, Marmion	LB	30	3.1	31° 48′ 32.4″	115° 43′ 1.2″	30	23	0.76	42	3	48.1	49.0	0.014	10
6	Fremantle fishing boat harbor	FB	30	3.8	32° 04′ 24.9″	115° 44′ 23.9″	30	27	0.90	46	1	47.9	47.5	−0.007	–
7	Parker Point, Rottnest Isl.	RPP	30	2.5	32° 01′ 28.2″	115° 31′ 45.6″	30	10	0.31	26	1	50.3	41.4	−0.240	–
8	Parmelia Bank east	D2	30	5.0	32° 08′ 07.8″	115° 43′ 8.4″	30	30	1.00	48	2	48.1	49.2	0.008	–
9	Carnac Island	CI	30	1.1	32° 07′ 12.20″	115° 39′ 54.3″	30	30	1.00	30	–	22.2	21.9	−0.036	–
10	Southern Flats, Cockburn Sound	SF	30	2.2	32° 15′ 3.84″	115° 42′ 22.4″	30	25	0.83	34	–	41.5	45.1	0.068*	–
11	Point Peron	PP	30	0.5	32° 16′ 18.60″	115° 41′ 22.6″	30	21	0.69	35	–	48.7	45.4	−0.048	–
12	Safety Bay, Warnbro Sound	SB	30	2.0	32° 18′ 19.14″	115° 42′ 13.6″	30	19	0.62	37	–	46.0	47.2	0.044*	–
13	Whalers Cove, Frenchmans Bay	FR	31	0.5–1.0	35° 05′ 29.8″	117° 56′ 57.7″	29	28	0.96	38	4	57.1	56.2	−0.018	–
14	Oyster Harbour	OH	31	1.0–2.0	34° 58′ 57.9″	117° 58′ 29.9″	30	13	0.41	33	3	48.7	48.4	−0.044	–
15	Waychinicup Inlet	WI	31	0.5–1.2	34° 53′ 36.9″	118° 19′ 58.8″	30	26	0.86	30	1	45.7	40.6	−0.105	–
16	Cheynes Beach	CB	31	1.2	34° 52′ 46.3″	118° 24′ 21.7″	30	14	0.45	18	–	14.5	16.7	0.095	–
17	Duke of Orleans Bay, Esperance	DO	31	0.5	33° 55′ 32.0″	122° 34′ 52.2″	27	15	0.54	51	6	74.1	64.0	−0.184*	5
South Australia (Flindersian)															
18	Goose Island, Spencer Gulf	GI	33	3.0–4.0	34° 27′ 15.9″	137° 21′ 58.2″	30	11	0.34	36	2	54.3	50.9	−0.052	23
19	Fitzgerald Bay, Spencer Gulf	SPG	33	0.9	32° 56′ 23.7″	137° 45′ 27.5″	29	16	0.54	52	1	51.2	59.2	0.107**	3
20	Ardrossan, St Vincent Gulf	AR	33	4.2	34° 25′ 29.6″	137° 55′ 33.5″	30	23	0.76	36	2	41.4	43.8	0.042*	3
21	Marion Bay, St Vincent Gulf	MA	33	2.0	35° 14′ 13.1″	136° 58′ 58.6″	30	17	0.55	39	2	57.0	53.4	−0.066	14
22	American River, Kangaroo Island	KI	33	1.1	35° 39′ 53.7″	137° 37′ 35.8″	29	27	0.93	46	4	53.8	52.8	−0.008	10
23	Nora Creina	NC	33 (34)	1.8	37°19′ 34.4″	139° 50′ 55.3″	30	8	0.24	22	–	45.8	39.1	−0.160	–
Victoria (Maugean)															
24	Saint Margaret Island, Corner Inlet	SMV	37	0.5	38° 39′ 02.1″	146° 48′ 21.7″	30	14	0.45	22	–	41.9	36.5	−0.124	–
25	Duck Point, Corner Inlet	DPV	37	1.0	38° 48′ 09.0″	146° 16′ 07.6″	30	12	0.38	24	–	39.8	37.4	−0.073	2
Tasmania (Maugean)															
26	Stony Point, Robbins Passage	TSP	35	1.0–2.0	40° 44′ 32.5″	144° 58′ 46.2″	30	10	0.31	20	–	41.1	34.4	−0.155	–
27	Sea Oak Point, Tamar River	TLB	35	0.4	41° 04′ 38.8″	146° 48′ 10.7″	30	28	0.93	25	–	49.2	45.1	−0.101	–
28	Low Head, Tamar River	TLH	35	0.2	41° 03′ 57.3″	146° 47′ 41.4″	29	28	0.96	33	–	40.5	43.6	0.046**	1
29	Little Musselroe Bay	TLM	35	3.0	40° 45′ 44.6″	148° 02′ 21.8″	30	8	0.24	23	–	51.4	41.8	−0.206	1
30	Fotheringate Beach, Flinders Island	TFB	37	1.5	40° 12′ 53.9″	148° 01′ 43.9″	30	26	0.86	29	–	43.7	40.4	−0.082	–
31	Deal Island, Kent Group	TDI	37	6.0	39° 28′ 25.5″	147° 18′ 38.4″	29	7	0.21	18	–	27.0	28.5	0.003	–
32	Hogan Island	THI	37	11.0	39° 12′ 35.7″	146° 59′ 46.4″	30	10	0.31	23	–	48.6	38.4	−0.250*	3
New South Wales (Peronian)															
33	Pambula Lake	PL	37	1.0–2.0	36° 58′ 32.1″	149° 53′ 37.3″	30	12	0.38	21	–	29.6	30.1	−0.001	2
34	Merimbula Lake	ML	37	1.0–2.0	36° 53′ 50.2″	149° 54′ 31.8″	30	9	0.28	17	–	39.5	32.2	−0.228	–
35	St Georges Basin	SG	38	1.0–2.0	35° 07′ 02.5″	150° 39′ 39.5″	30	21	0.69	17	–	24.9	24.9	−0.012	–
36	Jervis Bay	JB	38	1.0–2.0	35° 00′ 04.0″	150° 43′ 32.9″	30	3	0.07	15	–	29.6	17.9	−0.656	–
37	Port Hacking	PH	38	1.0–2.0	34° 04′ 00.7″	151° 07′ 48.2″	30	5	0.14	19	1	64.4	35.6	−0.675	–
38	Botany Bay	BB	38	1.0–2.0	34° 00′ 23.2″	151° 11′ 21.0″	30	7	0.21	13	–	11.1	12.1	0.044	–
39	Port Jackson (Sydney Harbour)	SH	38	1.0–2.0	33° 48′ 13.9″	151° 16′ 13.9″	30	7	0.21	19	–	31.7	28.5	0.007*	13
40	Pittwater	PW	38	1.0–2.0	33° 35′ 31.2″	151° 19′ 12.3″	30	3	0.07	12	–	7.4	6.2	−0.200	–
41	Brisbane Water	BW	38	1.0–2.0	33° 27′ 05.0″	151° 19′ 39.2″	30	3	0.07	16	–	44.4	27.2	−0.567	1
42	Lake Macquarie	LM	38	1.0–2.0	33° 02′ 59.8″	151° 38′ 49.1″	30	3	0.07	14	–	33.3	19.1	−0.675	–
43	Port Stephens	PS	38	1.0–2.0	32° 43′ 07.5″	152° 04′ 34.1″	30	7	0.21	14	–	6.3	17.5	0.497**	–
44	Wallis Lake	WL	38	1.0–2.0	32° 14′ 49.2″	152° 28′ 08.4″	30	2	0.03	13	–	38.9	20.8	−0.833	–
	Overall						1312	642	0.49	146	–	42.1	36.9	−0.138	170

*Note*: Significant deviation from Hardy–Weinberg equilibrium, *p < .05 and **p < .005.

Abbreviations: *N*, number of shoots genotyped; MLG, number of unique multilocus genotypes; *R*, clonal diversity, where *R* = (MLG‐1)/(*N*−1); Genetic diversity indices based on MLGs: Na, number of alleles; *p*[*i*], private alleles; *H*o, observed heterozygosity; *H*e, expected heterozygosity; *F*, Fixation Index; 3×, number of samples with at least one additional allele.

### 2.2 Genetic analyses

Clonal richness (*R* = (*G*−1)/(*N*−1)) was estimated for each meadow, where *G* = number of multilocus genotypes (MLGs) and *N* = number of samples (Dorken & Eckert, 2001). Additional genetic diversity indices within meadows were estimated based on MLGs: the total number of alleles (Na), number of private alleles (*p*[*i*]), observed heterozygosity (*H*_o_), expected heterozygosity (*H*_e_), and fixation index (*F*) using genalex 6.503 (Peakall & Smouse, 2012). Hardy–Weinberg equilibrium and linkage disequilibrium tests were performed using Genepop on the web 4.7 (Raymond & Rousset, 1995; Rousset, 2008). Patterns of allelic diversity were assessed visually (presence of consecutive‐sized alleles) to determine whether individual loci followed a stepwise model of mutation (addition or subtraction of single repeat units; Kimura & Ohta, 1978). Linear regressions were used to test the relationship between latitude (north to south along both west and east Australian coasts) and longitude (west to east across southern Australia) with measures of genetic diversity: clonal diversity (*R*), allelic diversity (Na), and expected heterozygosity (*H*_e_).

A principal coordinate analysis (PCoA) was implemented in genalex to visualize the genetic relationships among sampled meadows based on the complete dataset and repeated on a reduced dataset containing only unique MLGs. A Bayesian clustering approach was implemented in STRUCTURE v2.3.4 (Pritchard et al., 2000) to infer significant genetic clusters based on the complete dataset only. A reduced dataset (MLGs only) resulted in an uneven number of MLGs within sampled meadows due to extensive clonality in some meadows, which can create biases (Puechmaille, 2016). This approach identified the number of *K* clusters (or populations), assigned individuals to them, and identified admixed or migrant individuals. The analysis was performed for *K* = 1–44. A total of 10 independent runs were made for each value of *K* with 10,000 “burn‐in” and 100,000 replicates, using an admixture model with allele frequencies correlated across populations and without sampling locations specified as priors. Calculation of Δ*K* was used to infer the most likely number of clusters for 10 replicate runs for each *K* (Evanno et al., 2005) implemented using STRUCTURE HARVESTER v6.94 (Earl & von Holdt, 2012). A STRUCTURE bar plot was drawn using Structure Plot v2.0 (Ramasamy et al., 2014). Population differentiation was also estimated within each significant *K* cluster to determine regional structure, *F*_ST_ (Wright, 1943) and *D* (Jost, 2008), in genalex.

A hierarchical analysis of molecular variation (AMOVA) was performed in genalex to test for significance (at different spatial scales) among a priori recognized biogeographical provinces (Flindersian, Maugean, and Peronian), IMCRA inshore coastal provincial bioregions (29–38), and three “soft” seascape features as barriers to dispersal (Capes Upwelling, Great Australian Bight, and Bonney Upwelling; Figure 1), relative to genetic clustering. Components of variance were computed at these multiple levels, with significance based on 9999 permutations.

Overall and pairwise population differentiation was estimated using *F*_ST_ (Wright, 1943) and *D* (Jost, 2008) in genalex. Isolation‐by‐distance (IBD) relationships were assessed through the association between genetic differentiation (*F*_ST_ and *D*) and oceanographic distance (km) across the whole species range and by coastlines: latitudinal eastern (12 meadows; 32–37°S), western (12 meadows; 25–35°S), and longitudinal southern (20 meadows; 117–148°E) using Mantel tests (9999 iterations). Oceanographic distance was calculated using the “gdistance” package in R (3.4.4), which estimated the least‐cost (shortest) distance via water between GPS locations of all sampled meadows.

### 2.3 Mapping the historical and current distribution

Predictive modeling techniques were used to map the current distribution of *P. australis*, while the historical distribution of *Posidonia* habitat was created using depth as the primary distribution driver. Presence records for *P. australis* were downloaded from the Global Biodiversity Information Facility (GBIF: https://www.gbif.org/en/), of which 1153 reliable records were mapped using ArcGIS, after duplicates and points with errors in geo‐positioning were removed (Figure S1). A total of 23 environmental parameters that were potential predictors of *P. australis* occurrence were compiled from Bio‐ORACLE (Tyberghein et al., 2012; http://www.bio‐oracle.org/) and GeoScience Australia **(** https://www.ga.gov.au/data‐pubs) online data portals, at a spatial resolution of 5 arcmin. Highly correlated variables were removed to avoid excessive autocorrelation among predictors and reduce model overfitting based on variance inflation factor (VIF) values, which was calculated for each variable using the “vifstep” function in the “usdm” R package (Naimi et al., 2014). A threshold of VIF > 5 was used in this study as a conservative measure (Naimi et al., 2014). A final list of nine variables was used to build models (Table S1). Models were generated using five different methods and a model ensemble in the “sdm” R package (Naimi & Araújo, 2016). The following methods were implemented: boosted regression trees (brt), generalized linear model (glm), generalized additive model (gam), bioclim, and random forest (rf) (described in Turner et al., 2019). A fivefold cross‐validation procedure was implemented in each of the 10 replicate runs for each method. This resulted in 50 individual models for each method. Models were evaluated using AUC (area under the curve of a receiver–operator characteristic plot). Model ensembles were then created in the “sdm” package (Naimi & Araújo, 2016). Models with a test AUC > 0.7 were interpreted as a good performance (e.g., Coetzee et al., 2009; Swets, 1988), and were used to create the model ensemble which was then used to project *P. australis* probability of occurrence across temperate Australia. A conservative presence threshold of 0.7 was used to map the final distribution. The historical distribution for *P. australis* was predicted based on current knowledge of light requirements, bathymetry, and paleocoastline, with a potential distribution to a depth of 20 m.

## 3 RESULTS

### 3.1 Genetic diversity within meadows

Tri‐allelic genotypes were observed in some meadows (12.9% of samples; Table 1). These were reduced to diploid genotypes for all analyses as follows. Alleles that were not detected in a homozygous form, or were rare (*f* < 0.05), were removed. Identical, commonly occurring tri‐allelic genotypes were reduced to the same diploid genotype, so as not to alter the total number of MLGs (as per Sinclair et al., 2020).

MLGs were obtained for 1312 samples from 44 meadows throughout the range of *P. australis*, of which 642 were unique (*R* = 0.49). All meadows contained a set of unique MLGs, with the exception of northern range edge meadows on the west and east coasts. One MLG was shared between two meadows within the western gulf of Shark Bay (Useless Loop and Denham; ~22 km apart) and seven MLGs were shared among northern meadows on the east coast (Botany Bay to Wallis Lake; up to 268 km apart). Clonal diversity within meadows varied from a single MLG to all samples having unique MLGs (Table 1). Thirty‐two of 44 meadows were in Hardy–Weinberg equilibrium (Table 1). Six meadows had a significant excess of heterozygotes, and another six had deficits in heterozygosity.

Patterns of allelic diversity in Western Australian meadows were mostly consistent with a stepwise mutation model (SMM). There were large gaps in the presence of alleles (by locus) in southern and eastern Australian meadows relative to the west coast meadows. Private alleles were almost exclusively observed in western and southern Australian meadows. There was a highly significant relationship between clonal diversity *R* and inbreeding *F* across sampled meadows for the whole dataset (Spearman correlation, *r*_*s*_ = 0.63, *p* < .005) and based on MLGs only (Spearman correlation, *r*_*s*_ = 0.52, *p* < .001). However, the relationship was not linear, with meadows close to Hardy–Weinberg equilibrium once clonal diversity was above ~0.20.

**FIGURE 2:**
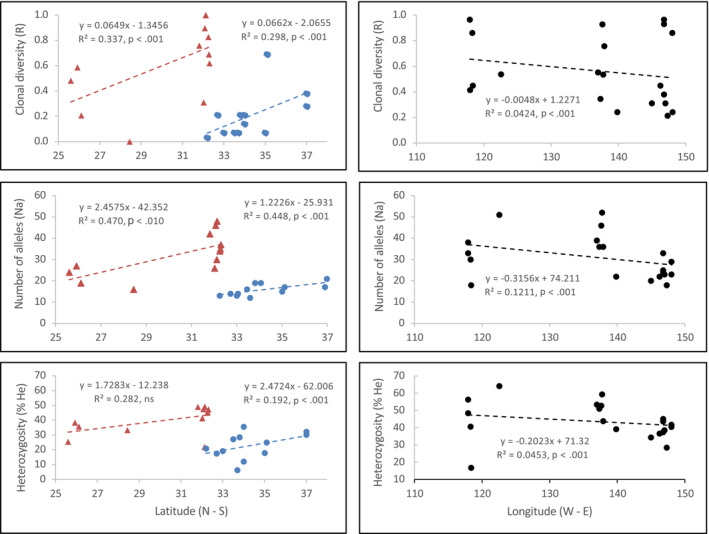
The relationship between three genetic diversity indices, clonal diversity (R) number of alleles (Na), and expected heterozygosity (He), and latitude down the west coast (red triangles) and east coast (blue circles) and longitude across the southern coastline (black circles) of Australia.

Higher clonality was observed toward northern range edges on both western and eastern coastlines. Genetic diversity increased with latitude for all three measures (*R*, Na, *H*_e_) down the west (12 meadows; *R* ^2^ = 0.337, *p* < .001; 0.470, *p* < .010; respectively, although *H*_e_ was not significant; 0.282, ns) and east coasts (12 meadows; *R* ^2^ = 0.298, 0.448, 0.192, all *p* < .001, respectively) (Figure 2). There was a weak, but significant, longitudinal relationship across southern Australia with all three genetic diversity measures (20 meadows; *R* ^2^ = 0.042, 0.121, 0.045; all *p* < .001; respectively; Figure 2).

### 3.2 Spatial genetic structure and biogeography

**FIGURE 3:**
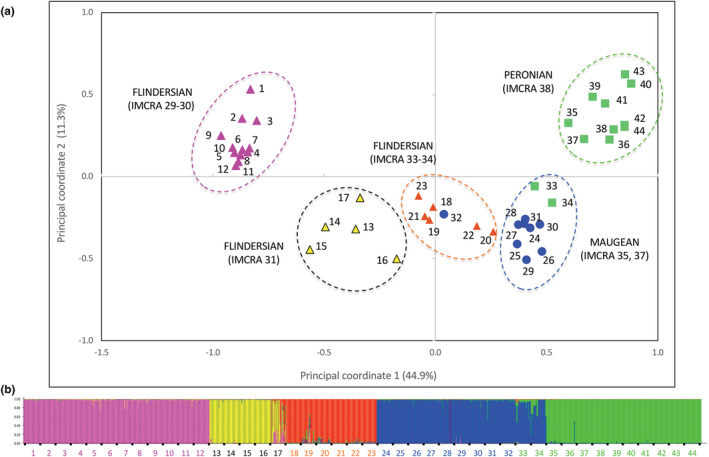
(a) Principal coordinates analysis (PCoA) of population means showing the relationship among 44 sampled *Posidonia australis* meadows. Meadows are coded according to their biogeographic province: Flindersian (triangles), Maugean (circles), and Peronian (squares), and IMCRA bioregion (29–38). Optimal *K* = 5 clustering from the STRUCTURE analysis is shown in color; west coast (pink), western‐south coast (yellow), central‐southern coast (orange), Bass Strait (blue), and east coast (green); (b) STRUCTURE analysis representing 44 sampled *P. australis* meadows from west to east coast (left to right). Each individual is represented by a single vertical line broken into segments, where segments are proportional to the membership coefficient for each of the population clusters (*K* = 5, mean Ln P(*K*) = −22775.2 ± 27.3). Five genetic clusters are: west coast meadows (pink; 1–12), south coast of Western Australia (yellow; 13–17), central‐southern Australian (orange; 18–23), Bass Strait (blue; 24–32), and east coast (green; 33–44) meadows.

Overall spatial genetic structure assessed using PCoA was similar using the complete dataset and MLGs only, despite the variable number of MLGs within meadows. The PCoA grouped meadows into five genetic clusters, consistent with spatial clustering of collection records in the GBIF database (Figures 3, S1). Each of the five genetic clusters corresponded to one of five endemic bioregions (IMCRA 29, 31, 33, 35, and 38). Meadows sampled within transitional bioregions (IMCRA 30, 34, and 37) clustered with an adjacent endemic bioregion. West coast meadows (IMCRA 29–30) were more tightly clustered together than those on the east coast (IMCRA 37, 38), despite a similar geographic distance. Meadows across the south coast of Western Australia (IMCRA 31) formed a separate cluster and were more similar to central‐southern Australian meadows (IMCRA 33, 34) than to those on the west coast. Meadows within the Bass Strait (IMCRA 35, 37) formed a tight cluster, with the exception of Hogan Island. The two southernmost east coast meadows, Pambula Lake and Merimbula Lake (33 and 34), were more similar to meadows within the Bass Strait compared to other east coast meadows.

A plot of the absolute second‐order rate of change of the likelihood distribution from the STRUCTURE analysis of the complete data showed a large peak at *K* = 2, which we attribute to the *K* = 2 conundrum (Janes et al., 2017). The “optimal” number of *K* clusters = 5 (Figures 3, S2) was consistent with the PCoA clustering. Regions of admixture were identified between geographically neighboring genetic clusters, with the exception of the west and south coast. There was significant differentiation among meadows within each of the five clusters (all comparisons *p* < .001): west coast (*n* = 12 meadows: *F*_ST_ = 0.268; *D* = 0.248), western‐south coast (*n* = 5 meadows: *F*_ST_ = 0.292; *D* = 0.410), central‐southern coast (*n* = 7 meadows: *F*_ST_ = 0.265; *D* = 0.349), Bass Strait (*n* = 10 meadows: *F*_ST_ = 0.228; *D* = 0.160), and east coast meadows (*n* = 10 meadows: *F*_ST_ = 0.310; *D* = 0.126).

At a higher level, genetic clustering was largely consistent with the three a priori marine biogeographic provinces (AMOVA, *p* < .001; Table 2). The Maugean (Bass Strait) and Peronian (east coast) genotypes were more closely related to each other than the Flindersian (Figure 3). Genetic structuring was also largely consistent with the finer‐scale IMCRA bioregions, accounting for 33% of variation (Table 2). Discrepancies were noted within the Flindersian and IMCRA bioregion 31 at the Capes region in southwestern Australia, while broad overlap between Maugean and Peronian provinces in southeastern Australia was better captured through IMCRA 35, 37, and 38 bioregions. Clustering among Flindersian and Maugean meadows was associated with three marine features, Capes Upwelling (31), Great Australian Bight (32), and Bonney Upwelling (34; all significant at *p* < .001; Table 2).

**TABLE 2:** Test of biogeographic hypotheses and three marine features: hierarchical analysis of molecular variance among sampled *Posidonia australis* meadows.

Source of variation	*F*‐statistic	df	SS	Variance component	Estimated variance	Total variation (%)	*p* value
Biogeographical provinces (Flindersian, Maugean, and Peronian)							
Among provinces	*F*_rt_	2	1702.2	851.1	1.0	26	>.001
Among meadows within provinces	*F*_sr_	41	2270.2	55.4	0.9	24	>.001
Among individuals within meadows	*F*_ST_	1268	1664.0	1.3	0.0	0	>.001
Within individuals	*F*_IS_	1312	2435.0	1.9	1.9	49	1.000
Total	*F*_IT_	2623	8071.4		3.8	100	>.001
Provincial bioregions (IMCRA 29–38, *n* = 7)							
Among provinces	*F*_rt_	6	2787.4	464.6	1.2	33	>.001
Among meadows within provinces	*F*_sr_	37	1185.0	32.0	0.5	15	>.001
Among individuals within meadows	*F*_ST_	1268	1664.0	1.3	0.0	0	>.001
Within individuals	*F*_IS_	1312	2435.0	1.9	1.9	52	1.000
Total	*F*_IT_	2623	8071.4		3.6	100	>.001
STRUCTURE analysis (genetic clusters, *n = 5*)							
Among provinces	*F*_rt_	4	2540.5	635.1	1.2	32	>.001
Among meadows within provinces	*F*_sr_	39	1431.9	36.7	0.6	16	>.001
Among individuals within meadows	*F*_ST_	1268	1664.0	1.3	0.0	0	>.001
Within individuals	*F*_IS_	1312	2435.0	1.9	1.9	51	1.000
Total	*F*_IT_	2623	8071.4		3.6	100	>.001
Capes Upwelling (within Flindersian and IMCRA 31)							
Among regions	*F*_rt_	1	360.9	360.9	0.9	26	>.001
Among meadows within regions	*F*_sr_	11	361.4	32.9	0.5	15	>.001
Among individuals within meadows	*F*_ST_	373	713.3	1.9	0.0	0	>.001
Within individuals	*F*_IS_	386	791.5	2.1	2.1	59	0.997
Total	*F*_IT_	771	2227.1		3.5	100	>.001
Great Australian Bight (IMCRA 32)							
Among regions	*F*_rt_	1	201.9	201.9	0.5	14	>.001
Among meadows within regions	*F*_sr_	8	400.7	50.1	0.8	23	>.001
Among individuals within meadows	*F*_ST_	284	567.3	2.0	0.0	0	>.001
Within individuals	*F*_IS_	294	657.5	2.2	2.2	63	1.000
Total	*F*_IT_	587	1827.4		3.6	100	>.001
Bonney Upwelling (IMCRA 34)							
Among regions	*F*_rt_	1	227.2	227.2	0.5	15	>.001
Among meadows within regions	*F*_sr_	13	443.8	34.1	0.5	18	>.001
Among individuals within meadows	*F*_ST_	431	697.0	1.6	0.0	0	>.001
Within individuals	*F*_IS_	446	905.5	2.0	2.0	67	1.000
Total	*F*_IT_	891	2273.5		3.0	100	>.001

**FIGURE 4:**
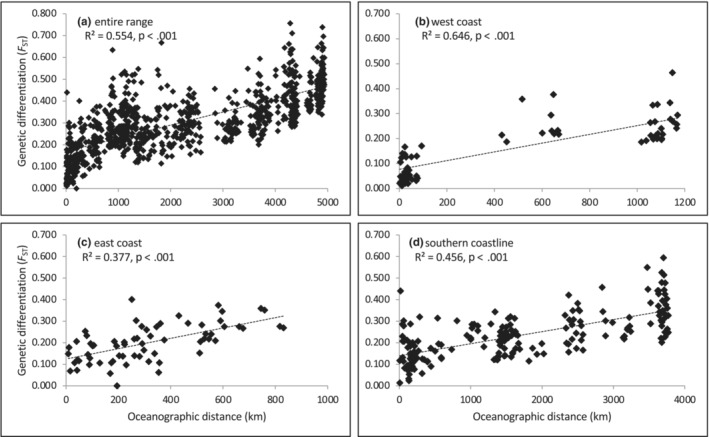
Isolation‐by‐distance relationship between genetic differentiation (*F*_ST_) and oceanographic distance (km) for *Posidonia australis* meadows sampled from (a) across the entire range; (b) west coast; (c) east coast; and (d) southern coastline. Regression lines are shown for each coastline, with *R* ^2^ and *p* values indicating the strength and significance of the relationship.

Overall genetic differentiation across the species range was high (*n* = 44 meadows: *F*_ST_ = 0.499; *D* = 0.527). There was significant pairwise differentiation among all meadows (*F*_ST_ = 0.021–0.736; *D* = 0.020–1.000; *p* < .001, Table S2); with the exception of three pairs, two high‐diversity meadows in close geographic proximity (Cockburn Sound—FB/D2, *F*_ST_ = 0.011; Tamar River estuary along the north coast of Tasmania—TLB/TLH, *F*_ST_ = 0.014) and highly clonal northern range edge meadows on the east coast which are 190 km apart (LM/WL; *F*_ST_ = 0.000). There was a highly significant IBD relationship between pairwise oceanographic distance and genetic differentiation across the species range (*n* = 44 meadows: Mantel tests *R* ^2^ = 0.554 (*F*_ST_), 0.805 (*D*)), with latitude along the western (*n* = 12: *R* ^2^ = 0.646 (*F*_ST_), 0.787 (*D*)) and eastern (*n* = 12: *R* ^2^ = 0.377 (*F*_ST_), 0.605 (*D*)) coastlines, and longitude across southern Australia (n = 20: R^2^ = 0.456 (*F*_ST_), 0.708 (*D*)) (all *p* < .001; Figure 4a–d).

### 3.3 Historical and current distribution

**FIGURE 5:**
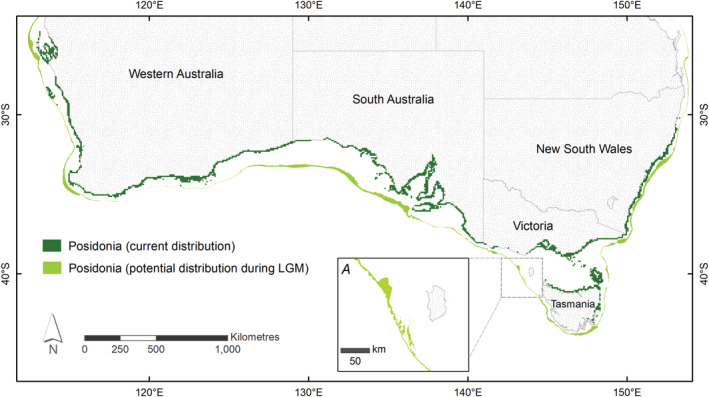
The modeled current (dark green) and historical distribution of *Posidonia* at the Last Glacial Maximum (c. 120 m below present sea level, light green) approximates the location of the Continental shelf margin. Inset A. Larger paleomeadows were present along the margins of the Otway Shelf.

The historical and current distribution models predict mostly continuous distributions across the southern half of the Australian coastline (Figure 5). The distribution of *Posidonia* habitats at the LGM (120 m below current sea level) shows potential for widespread fringing meadows, restricted to the edge of the contemporary continental shelf. The shallow continental shelf habitat available to benthic species including seagrass was less than the present day, with steep shelf edges reducing the total area to fringing meadows across much of the range. Consequently, the spatial extent of the modeled current *P. australis* distribution is about 110,000 km^2^, while the extent of the modeled historical distribution during the LGM was around 80,000 km^2^. The historical range showed greater northward range extensions on west and east coasts than the current distribution. The historical distribution has a very similar longitudinal range to the contemporary distribution, with the exception of what is currently the Bass Strait, which was entirely above sea level at the LGM. The presence of a large, hypothesized paleomeadow on the edge of the Otway Shelf was supported (originally proposed by Waters & Roy, 2003), from which the Bass Strait would have been initially recolonized (Figure 5 inset A).

## 4 DISCUSSION

Hierarchical patterns of range‐wide spatial genetic structure in *P. australis* broadly reflected the marine biogeographic provinces and IMCRA bioregions for southern Australia (Bennett & Pope, 1953; Commonwealth of Australia, 2006; Last et al., 2010). However, a significant deviation from both biogeographic frameworks occurred within the Flindersian Province and IMCRA southwest bioregion (31), where historical and contemporary conditions create an effective barrier to gene flow. Our results were consistent with previous observations (e.g., Ayre et al., 2009; Nielsen et al., 2021; Teske et al., 2017; Waters et al., 2010) that southern hemisphere cool‐temperate marine species have complex biogeographic histories in which historical and contemporary information on distribution, oceanography, climate, ecology, and life history are required for interpretation of spatial genetic structure. Our combination of historical and contemporary predictive mapping and population genetic data suggests how multiple seascape drivers have influenced the capacity of *P. australis* to effectively track sea level changes associated with natural climate cycles. Three known marine features, Capes Upwelling, Great Australian Bight, and Bonney Upwelling, also acted as barriers to dispersal in *P. australis*. The most genetically diverse meadows, situated in western and central southern Australia (IMCRA 31, 33), are located in a region recognized as the origin of diversification for Australian *Posidonia*, and coincide with high species diversity and endemism (Carruthers et al., 2007; Kendrick et al., 2009; Langlois et al., 2012).

### 4.1 Long‐term stability and broad biogeographic patterns

The combination of predictive mapping and/or population genetic assessments from multiple studies (e.g., Teske et al., 2017, 2018; Nielsen et al., 2021; Assis et al., 2022; this study) leads us to propose several broad biogeographical hypotheses for southern hemisphere temperate marine species: (1) strong latitudinal gradient in intraspecific diversity (highest diversity in southern populations); (2) past distributions extended into lower latitudes (more northerly distribution); (3) widespread distributions of coastal shelf species persisted during the LGM (not distinct, isolated refugia, as proposed by Waters and Roy (2003)); and (4) important role of “soft” oceanic features such as currents and upwellings, as shared historical and contemporary barriers to genetic connectivity. These are in striking contrast to equivalent latitudes in the northern hemisphere where glaciation caused marine taxa to retreat to periglacial and southern refugia (Maggs et al., 2008). For example, within the Mediterranean, paleodistributions predicted from ecological niche modeling for *Posidonia oceanica* at the LGM identified three putative refugia in the southern Mediterranean, western, central, and eastern locations (Chefaoui et al., 2017). These Pleistocene refugia were consistent with spatial genetic variation supporting a hypothesis for a secondary contact zone in the (central) Siculo‐Tunisian Strait between meadows in the eastern and western basins (Arnaud‐Haond et al., 2007; Serra et al., 2010). A Pleistocene legacy of significant genetic structure among the Atlantic and Pacific Ocean meadows of the seagrass, *Zostera marina*, also highlights the historical influence on modern marine ecosystems (Duffy et al., 2022).

The largely similar widespread historical and current *Posidonia* distributions suggest shared habitat connectivity over geological timescales (Miocene to present), with common seascape features influencing distributions and genetic structure. Two notable historical differences included range extensions into lower latitudes and the Bassian Landbridge (Figures 1 and 5). This long‐term persistence in habitat connectivity is likely driven by a stable Australian continental shelf and offshore boundary currents creating an environment that favored the persistence of widespread shallow‐water benthic communities. Extended periods of lower sea levels reduced the continental shelf area available for benthic marine ecosystems (Heap & Harris, 2008; James & Bone, 2011; Williams et al., 2018) and left a record of submerged paleoshorelines (Brooke et al., 2017; James & Bone, 2011). Given the high light requirement of *Posidonia*, the shallow, more gently sloping continental shelf margins would have likely retained large, well‐connected meadows, as seen in current distributions and supported by the historical distribution model. Contemporary shelf habitats were then recolonized by widespread seagrass meadows tracking rising sea levels. For example, genetic data for *P. australis* (Figure 2; Sinclair et al., 2016) suggest initial recolonization of the Bass Strait from the upper slope of the Otway Shelf to the west, which was also found for a sea star, *Coscinasterias muricata* (Waters & Roy, 2003). The secondary contact or admixture zones between southern and east coast meadows are evidence of this biogeographical barrier, which has been present on multiple occasions through millennia (>67 m below current sea level; Lambeck & Chappell, 2001).

In striking contrast to the general finding of an inverse relationship between genetic diversity and latitude for Australian marine species (Pope et al., 2015), we found that genetic diversity increased with increasing latitude for *P. australis* along both temperate western and eastern Australian coastlines. The significant latitudinal relationship occurred despite different continental shelf topologies, prevailing currents, and habitat availability. *Posidonia australis* meadows along the west coast are more exposed on a wide, open shelf, while east coast meadows are largely contained within river estuaries and coastal bays along a narrow shelf. Intraspecific diversity gradients can be difficult to predict (Lawrence & Fraser, 2020), however, for coastlines that have been stable for millennia, such as Australia and west Africa, significant latitudinal gradients in genetic diversity may be a feature within temperate environments (e.g., Assis et al., 2022).

Spatial patterns of genetic structure across the world's longest boundary circulation, which traverses Australia's southern coastline (Middleton & Bye, 2007), were weakly associated with two habitat‐forming species, *P. australis* (this study) and the kelp *Ecklonia radiata* (Coleman et al., 2011). *Ecklonia radiata* appeared to be correlated with the strength of boundary currents (Coleman et al., 2011), while the pattern in *P. australis* was more complex. The weak IBD association in *P. australis* was disrupted by higher genetic diversity in central‐southern Australia meadows (IMCRA 33), an observation also noted in seadragons *Phyllopteryx taeniolatus* (Wilson et al., 2017) and *Phycodurus eques* (Stiller et al., 2021). Sparse sampling across southern Australia has often limited wider conclusions (Coleman et al., 2011; Kassahn et al., 2003; Stiller et al., 2021; Wilson et al., 2017), however, the higher genetic diversity in *P. australis* meadows may be associated with a change in ploidy, as recently detected in Shark Bay meadows (Edgeloe et al., 2022).

### 4.2 Provincial bioregions and spatial genetic structure

Range‐wide spatial genetic structure in *P. australis* was largely associated with the IMCRA provincial bioregions (Commonwealth of Australia, 2006; Last et al., 2010), however, there were discrepancies. One discrepancy occurred at the junction between the Indian (west coast) and Southern Oceans in the Cape Naturaliste‐Leeuwin region. Here, meadows on either side of the Capes Region, within the Flindersian province and southwest bioregion (IMCRA 31), comprised distinct genetic clusters. The break occurred in a region more commonly associated with species turnover for inshore species (O'Hara & Poore, 2000; Smale et al., 2010). An offshore upwelling drives the nearshore Capes Current northward along the Western Australian coast, creating a physical and temporal barrier to gene flow (see Hanson et al., 2005). This seasonal upwelling occurs during the Austral Spring/Summer, coinciding with fruit release for reproductive meadows. Significant genetic structure was also reported in this region for *E. radiata* (Vranken et al., 2021).

A second region of discrepancy occurred in southeastern Australia, where untangling the complex genetic signature within the Bass Strait is challenging. The genetic signature of a historical disjunction in central Bass Strait (Wilsons Promontory within IMCRA 37) is shared among many marine invertebrate taxa, and sequence divergence estimates pre‐date the LGM (reviewed in Ayre et al., 2009; Teske et al., 2017). The genetic signature in *P. australis* closely reflected the Bassian drainage divide indicated through the island chain in eastern Bass Strait (see Buckley et al., 2021), the last points of connection between Tasmania and mainland Australia with sea level rise following the LGM. However, the Corner Inlet meadows to the east of Wilsons Promontory (also within IMCRA 37) were clustered with Bass Strait islands and mainland Tasmanian meadows (IMCRA 35). This discrepancy likely reflects temporal changes in dispersal connectivity associated with inundation following the LGM, noting these meadows were not connected via hydrodynamic modeling of current seed dispersal (Sinclair et al., 2016).

The *P. australis* meadows associated with transitional bioregions (IMCRA 30, 32, 34, and 37) contained genetic signatures of admixture and/or restricted gene flow associated with “soft” biogeographic or oceanographic barriers to dispersal, geomorphic features and/or largely reflected a naturally fragmented distribution. Admixture coincided with the Great Australian Bight (IMCRA 32) and southeast transitions (IMCRA 37). The high levels of genetic diversity and admixture between western and central southern clusters support a more continuous distribution for *P. australis* across southern Australia, as our distribution models suggest. The Great Australian Bight is not widely reported as a temperate marine barrier (see Teske et al., 2017), so while admixture has been observed among populations on either side of the Great Australian Bight (e.g., Stiller et al., 2021; this study), further exploration and sampling of such remote coastlines are required to increase our understanding of marine connectivity across southern Australia. Strong seasonal variability and reversal of flow direction occur (i.e., also east to west; Duran et al., 2020) in the inshore coastal currents driving *Posidonia* dispersal along the southern Australian coastal shelf. This fits with contemporary ecological observations by Kendrick et al. (2009), who noted that marine biota in the Recherche Archipelago, east of Esperance, have a greater affinity to the cool‐water assemblages of central‐southern Australia than to the west coast, thus supporting connectivity across the GAB transition zone (IMCRA 32). Phylogenetic structuring in leafy seadragons, *Phycodurus eques* (Stiller et al., 2021), also supports this relationship by suggesting that western regions were recolonized from the east during post‐LGM inundation of the Continental shelf.

*Posidonia australis* meadows are highly fragmented along the exposed coastlines within the Southwest and Bass Strait transitions (IMCRA 30, 34). The western Bass Strait transition (IMCRA 34) contains a seasonal upwelling, Bonney Upwelling (Kämpf et al., 2004) which also appears to restrict gene flow between central‐southern Australia (IMCRA 33) and Bass Strait (IMCRA 35). Admixture among *P. australis* meadows across the southeastern transition (IMCRA 37) was reported in species with low and high dispersal capabilities (e.g., Miller et al., 2013; Stoessel et al., 2020; Waters & Roy, 2003), so there is no consensus on barriers to gene flow in this region. For example, multiple features occur including 90‐mile beach, a significant change in geomorphic structure (figure 9 in Heap & Harris, 2008), and the winter 14°C sea surface isotherm (O'Hara & Poore, 2000). The spatial genetic structure reflects the distance between available habitats, as per Ayre et al. (2009), as well as the complex coastlines and life‐history traits which can create an appearance of localized “chaotic genetic patchiness” due to genetic drift and temporal variation in collective dispersal events (Broquet et al., 2013; Johnson & Black, 1982; Sinclair et al., 2014). Some seagrass species are particularly well adapted for inshore sea surface dispersal of floating fruits and/or reproductive plant parts (e.g., Hernawan et al., 2017; Sinclair et al., 2016). Contemporary dispersal by *P. australis* fruits is influenced by daily shifts in local prevailing winds, with distances of 100–150 km representing the upper limit for dispersing (Ruiz‐Montoya et al., 2012, 2015; Sinclair et al., 2018). The range‐wide association between genetic and geographic distance in *P. australis* is congruent with a widespread species displaying a regional dispersal capacity, the scale of which is broadly consistent with spatial genetic patterns found in other widespread seagrasses (reviewed in Kendrick et al., 2012).

### 4.3 Implications for the future of climate change

Overall, our combination of predictive distribution mapping and analysis of spatial genetic structure has allowed us to infer how historical and contemporary seascape features have influenced the capacity of *P. australis* to effectively track sea level changes associated with natural climate cycles over millennia via clonal and sexual reproduction. The similarities between historical and current distributions were consistent with shared biogeographical features and long‐term resilience in *P. australis*. However, recent increases in ocean temperatures and acidification associated with global warming create a more stressful environment, with many temperate species already on the move, retreating to deeper waters, higher latitudes, or becoming locally extinct (Pecl et al., 2017). There is potential for a southward range expansion of *P. australis* down Tasmania's east coast into suitable habitat, as identified in our modeling. Projections for *P. oceanica* in the Mediterranean suggest it will no longer be able to inhabit its' current range, with a 75% loss of suitable habitat by 2050 and functional extinction by 2100 (Chefaoui et al., 2017). Similarly, the physiological predictions for survival of *P. australis* to increasing temperatures along the west coast of Australia suggest a range contraction of between 200 and 400 km by 2100 (Hyndes et al., 2016). Whole‐genome duplication through polyploidy in *P. australis* has apparently increased thermal and salinity tolerance in extreme environments and enabled colonization of Shark Bay waters post‐LGM (Edgeloe et al., 2022). This is an example of the evolutionary significance of polyploidy and shows how species and populations have and will continue to evolve and adapt to stressful environments (Van de Peer et al., 2021). Further research is required on the role of hybridization and Holocene range expansions into the warm, hypersaline environments in the upper gulf region of central‐southern Australia. Our current study shows a historic resilience to climate change in *P. australis* and suggests stability in the current IMCRA bioregions for interpreting biogeographic patterns. However, more detailed sampling across a range of taxa will be required to resolve many of the discrepancies, in which a nested mesoscale framework may better capture diversity and spatial genetic structure and be a preferred framework for managing biodiverse ecosystem structure.

## AUTHOR CONTRIBUTIONS

**Elizabeth A. Sinclair:** Conceptualization (lead); data curation (equal); formal analysis (equal); funding acquisition (equal); investigation (equal); methodology (equal); visualization (equal); writing – original draft (equal); writing – review and editing (equal). **Renae K. Hovey:** Conceptualization (equal); formal analysis (equal); methodology (equal); visualization (equal); writing – review and editing (equal). **Siegfried L. Krauss:** Formal analysis (equal); funding acquisition (equal); writing – review and editing (equal). **Janet M. Anthony:** Data curation (equal); methodology (equal); writing – review and editing (equal). **Michelle Waycott:** Funding acquisition (equal); writing – review and editing (equal). **Gary A. Kendrick:** Conceptualization (equal); funding acquisition (equal); investigation (equal); project administration (equal); writing – review and editing (equal).

### OPEN RESEARCH BADGES

This article has earned an Open Data badge for making publicly available the digitally‐shareable data necessary to reproduce the reported results. The data is available at https://datadryad.org/stash/share/PpQGkzGdMRNsQEqqAnLaOKvYRjzjWX4‐DIQb5qjgmdU.

## Supporting information


Figure S1.

Table S1.

Table S2.

## Data Availability

Raw genotype data will be available from Dryad: https://datadryad.org/stash/share/PpQGkzGdMRNsQEqqAnLaOKvYRjzjWX4‐DIQb5qjgmdU.
