# Supplementary material for: Historic and contemporary biogeographic perspectives on range‐wide spatial genetic structure in a widespread seagrass
Source: Ecol Evol. 2023 Mar 19;13(3):e9900. doi: 10.1002/ece3.9900 (PMC10025079; doi:10.1002/ece3.9900)
Supplement: Supplementary file 1 — Figure S1. [file ECE3-13-e9900-s001.docx]

**Supporting Information Fig. S1.** Location of 1,153 records for *P. australis* downloaded from the Global Biodiversity Information Facility (GBIF: <https://www.gbif.org/en/>). Current records suggest a naturally fragmented distribution across its range.

**Supporting Information Fig. S2.** STRUCTURE analysis inferring population structure across sampled *P. australis* meadows from *K* = 1 - 44 with vertical bars indicating standard deviation from ten replicates per run.
