# Supplementary material for: Historic and contemporary biogeographic perspectives on range‐wide spatial genetic structure in a widespread seagrass
Source: Ecol Evol. 2023 Mar 19;13(3):e9900. doi: 10.1002/ece3.9900 (PMC10025079; doi:10.1002/ece3.9900)
Supplement: Supplementary file 2 — Table S1. [file ECE3-13-e9900-s002.pdf]

Supporting Information Table S1. Nine environmental variables used in the predictive model for current *Posidonia* distribution.

**Acronym**	**Variable**	**Units**
Currmax	Max current velocity at bottom	m/s
Currmean	Mean current velocity at bottom	m/s
Depth	Water depth	m
Nitrate	Nitrate	µmol/L
Salinity	Salinity	PSU
Sstmax	Max sea surface temperature	°C
Sstmean	Mean sea surface temperature	°C
Sstmin	Min sea surface temperature	°C
Sstrange	Range of sea surface temperature	°C

Layers downloaded from Bio-ORACLE with Depth variable downloaded from GeoScience Australia. All layers were resampled to a resolution of 5 arcmin (~9.2 km). The layers were constrained by a depth mask to a depth of 100 m in order to exclude deep waters with unsuitable seabed conditions for the modelled organism.
